# Alexinomia: The fear of using personal names

**DOI:** 10.3389/fpsyg.2023.1129272

**Published:** 2023-03-17

**Authors:** Thomas Ditye, Natalie Rodax, Lisa Welleschik

**Affiliations:** ^1^Faculty of Psychology, Sigmund Freud University, Vienna, Austria; ^2^Association for Psychotherapy, Counselling, Supervision and Group Facilitation, Vienna, Austria; ^3^Institute for Person-Centered Studies, Vienna, Austria

**Keywords:** social anxiety, fear, attachment, identity, names, addressing, alexinomia

## Abstract

**Introduction:**

Preliminary research based on everyday observations suggests that there are people, who experience severe fear when addressing others with their personal names. The aim of this study was to explore the extent to which this hitherto little-known psychological phenomenon really exists and to investigate its characteristic features, considering the everyday experience of not being able to use names and its impact on affected individuals and their social interactions and relationships.

**Methods:**

In this mixed-methods study based on semi-structured interviews and psychometric testing, 13 affected female participants were interviewed and evaluated using self-report measures of social anxiety, attachment-related vulnerability, and general personality traits. An inductive content analysis and inferential statistical methods were used to analyze qualitative and quantitative data, respectively.

**Results:**

Our findings show that affected individuals experience psychological distress and a variety of negative emotions in situations in which addressing others with their name is intended, resulting in avoidance behavior, impaired social interactions, and a reduced quality of affected relationships.

**Discussion:**

The behavior can affect all relationships and all forms of communication and is strongly linked to social anxiety and insecure attachment. We propose calling this phenomenon *Alexinomia*, meaning “no words for names”.

## 1. Introduction

*“It has always been like that, as long as I can remember. I couldn’t address others with their names, and it took extreme efforts to try. I became really conscious of it, when I met my husband. I wanted to address him by name, but I could not do it. That’s when I realized that it’s a problem, that I can’t say other people’s names.”*

In this article, we present an unknown psychological phenomenon characterized by *knowing* a name but *being unable to use it in personal communication*. For affected individuals, it is impossible to say, for example, “Good morning, Maria.” or “Armin! Great to see you.” They experience acute anxietiy and a variety of other negative emotions in social situations in which using a personal name is intended. We refer to this undocumented psychological phenomenon as *Alexinomia.* Alexinomia is a compound of the Greek loanwords á (a-, “not”) + λέξεις (*léxis*, “words”) + óν*o*μα (ónoma, “name”) and literally means “no words for names.”

Personal names—in the context of this article defined as first names, given names, and forenames, rather than last names, family names—are of substantial social importance (Horne, 1986). We use personal names to address and to greet others, to personalize conversation, and to call people over a distance. Names can be indicative of a person’s gender (Cassidy et al., 1999), age group (Lansley and Longley, 2016), socio-economic status (Bloothooft and Onland, 2011), and often have a literal or connotative meaning that people identify with (Brennen, 2000). In many situations, personal names are deliberately used to show respect, affection, and to create closeness. In this way, names determine our social interactions and, on an intra-individual level, are a fundamental factor for the sense of personal identity (Dion, 1983; Alford, 1987; Pilcher, 2016; Watzlawik et al., 2016).

The significance of using names in personal conversations has been highlighted in research in various fields. In education it has been claimed that teachers knowing and using the students’ names creates an atmosphere of trust and community in the classroom that facilitates learning (Glenz, 2014). It has been argued that calling students by their names makes them feel cared about and taken seriously (Syverud, 1993). Deliberately using first names to establish connection and equality in a relationship is a well-established technique in sales and in other persuasion-based occupational areas (DeCormier and Jackson, 1998). Furthermore, the importance of using names has been discussed in psychoanalytic texts in the context of the unpleasant effects of forgetting someone’s name on the affected individual (Murphy, 1957).

From a psychological perspective, the subjective experience of fear related to addressing others by name was preliminarily described by Welleschik (2019). Based on personal observations and unsystematic online research, Welleschik (2019) portrayed the case of a young woman who was unable to say her partner’s name. The preliminary findings provided by this research suggest that being unable to address others by name has severe consequences for personal and professional life. With partners, friends, and family, not saying their names may be considered impersonal or even rude and can create a social distance that is usually neither intended nor appreciated. In other situations, such as work meetings or at school, being unable to name others can heavily limit someone’s options, for example, when it comes to speaking up in groups, attracting attention, or to nuance conversation in a professional manner. The impact of this behavior on relationships is significant and concerns both the primarily affected individuals and the people around them. In severe cases, affected persons report to have never used their spouse’s name or have not said anyone’s name in many years.

Welleschik (2019) made references to the reports of other cases of affected individuals found online, which in the meantime have been confirmed by a systematic analysis of 257 online forum and blog posts by people expressing a problem in the specific social context of addressing others with their names (Bergert et al., unpublished data). Given the large scope of these materials, it is surprising that this distinct phenomenon is still undocumented. So far, there is no published scientific evidence based on systematic, empirical research showing that alexinomia really exists and describing how it affects those concerned and their relationships. The number of affected individuals is unknown, and so are the origins and causes of the problem.

To address the most fundamental open questions related to alexinomia, an explorative mixed-methods study was employed that expanded qualitative (i.e., interview) with quantitative (i.e., psychometric) data. To identify the experiential grounds and constituting factors of alexinomia, we employed a descriptive approach to inquiry using qualitative semi-structured interviews and inductive content analysis.

In addition, a psychometric test battery, including a set of standardized psychological self-report questionnaires, was administered to test for links of alexinomia with already known psychological constructs. Given the social importance of names and the social nature of the situations in which alexinomia seems to occur most frequently, social anxiety was considered a likely candidate to underly the problem. The role of names in identity formation (Watzlawik et al., 2016) and the differentiation of oneself and others (Palsson, 2014) led us to assume that these factors are likely to play a crucial role in alexinomia. Further, alexinomia-related symptoms seem to become more severe the closer the affected relationship is. Therefore, it seemed likely that problematic name saying could originate from attachment-related vulnerabilities and insecurities. Additionally, a test of general personality traits (i.e., big 5) was administered to test for general peculiarities that might arise in the personality structure of individuals with alexinomia. See Table 1 for a complete list of all instruments.

**TABLE 1:** Psychometric measures.

Instrument	Psychological constructs measured	Number of subscales/Cutoffs	Subscales	Number of items	References
Big Five Inventory 2 (BFI-2)	Big Five personality traits	Five subscales	Extraversion Agreeableness Conscientiousness Negative emotionality Open mindedness	60	Danner et al., 2016; Soto and John, 2017
Social Interaction Anxiety Scale (SIAS)	Social interaction anxiety	One cutoff	Social anxiety	20	Mattick and Clarke, 1998; Stangier et al., 1999
Differentiation of Self Inventory (DSI)	Differentiation of Self	Four subscales	Emotional reactivity Emotional cutoff I-Position Fusion with others	46	Maß et al., 2019; Skowron and Friedlander, 1998
Experience in Close Relationships (ECR-RD12)	Experience in close relationships	Two subscales	Attachment-related anxiety Attachment-related Avoidance	12	Brenk-Franz et al., 2018; Wei et al., 2007
Vulnerable Attachment Style Questionnaire (VASQ)	Vulnerable and insecure attachment	Three cutoffs	Vulnerability of attachment Insecurity of attachment Proximity seeking	22	Bifulco et al., 2003; Reck, 2009

By expanding qualitative data with already known psychological attributes using quantitative parameters, our intention was to provide an as comprehensive a picture of the phenomenon as possible. The method and results sections are therefore divided into qualitative and quantitative procedures and results. In the discussion, we draw links between the different levels of data and give a summarizing interpretation that allows a first systematic conceptualisation of alexinomia.

## 2. Materials and methods

### 2.1. Participants

Thirteen German-speaking women with a mean age of 28.1 years (range: 18–40 years) participated in the study. Participants were recruited from a patient database created by one of the authors (L.W.) in response to letters from affected individuals that were received *via* the author’s personal website^1^ over the past few years. At the time of data collection, the database included about 80 entries of affected individuals from all genders and many countries. Most contacts belonged to the group of young, female, German-speaking adults. Hence, for the purpose of this study, we recruited participants from this largest group of available contacts. The sample size was determined after iteratively analysing the contents of the first interviews. Once the central conceptual categories had emerged and started reappearing throughout most of the interviews, we stopped the sampling process.

Out of these 13 participants, 11 indicated noticing the problem of not being able to use names daily or multiple times per day. Nine participants reported noticing it for the first time as a child or teenager. The degree of psychological strain caused by the problem was rated as 5.7 on average (min = 3; max = 10; scale: 0–10). A summary of participants’ socio-demographic data and descriptive data with regards to the symptoms of alexinomia are shown in Table 2.

**TABLE 2:** Socio-demographic data.

Subject no.	Age	Gender	Nat.	Relationship status	Education	Frequency of symptoms^a^	First occurrence^b^	Psychological strain^c^	Therapy interest^d^
1	31	Female	DE	Married	High school	Daily or more	Age 13; first relationship	7	Yes
2	35	Female	DE	In relationship	Middle school	Daily or more	Age 14; first relationship	6.5	Yes
3	21	Female	CH	Married	High school	Daily or more	Childhood	10	Unsure
4	25	Female	DE	Single	High school	Daily or more	Age 12	5	N/A
5	30	Female	DE	Single	Higher education	A few times a month	N/A	3	Yes
6	30	Female	RO	Married	Middle school	Daily or more	first relationship	7.5	N/A
7	30	Female	DE	In relationship	Higher education	Daily or more	Age 15	4.5	N/A
8	22	Female	AT	Single	Higher education	Daily or more	Age 20	6	Yes
9	23	Female	DE	Single	High school	Daily or more	Age 15	5	N/A
10	35	Female	CH	In relationship	N/A	A few times a week	Age 28	7	Unsure
11	18	Female	DE	Single	Middle school	Daily or more	Always	3	Yes
12	25	Female	DE	In relationship	High school	Daily or more	Childhood	6	Yes
13	40	Female	DE	In relationship	High school	Daily or more	Age 21	3	N/A

^a^Frequency of symptoms: how often do you experience any symptoms related to alexinomia?

^b^First occurrence: when did you notice any difficulty related to saying names for the first time?

^c^Psychological strain: subjective degree of psychological strain related to alexinomia on a scale from 0 to 10.

^d^Therapy interest: would you be interested in therapy to treat the problems related to alexinomia or are you already in therapy?

### 2.2. Interviews

Semi-structured interviews were conducted with all participants to explore in depth their everyday lived experience in relation to using names. An interview guide including a set of open-ended questions was prepared before data collection that was flexible as to the sequence of questions and adapted according to the responses of the interviewees. The interview guide was structured into three main topics: (1) General experiences with the phenomenon of having difficulty addressing someone by their name, (2) coping strategies and (3) self-theories on alexinomia-related symptoms. This interview structure ensured that participants could develop their narratives openly to maximize our understanding of the phenomenon by allowing participants’ own frame of reference and concepts to unfold naturally (Willig, 2013). Where necessary, the interviewers posed individualized follow-up questions to be responsive to the interviewees and encourage them to expand on their experiences, feelings and/or self-theories. Follow-up questions were either probing questions aimed at further elaboration or specifying questions.

At the end of the interview, the interviewers asked for the relevant socio-demographic data that would allow for a sufficient contextualization of the interview data. The following factors were addressed: age, gender, partnership, the highest level of education, occupation, frequency of experiencing difficulties with using names (*How often do you experience these difficulties?*), history (*When did it first occur?*), personal psychological strain (0 to 10), and interest in a therapy to treat the problem.

### 2.3. Psychometric measures

The following psychometric instruments were used to measure personality factors that might be linked to alexinomia. We used the German versions of all listed scales: (1) Big Five Inventory (BFI-2; Soto and John, 2017): The BFI-2 assesses the Big Five personality domains Extraversion, Agreeableness, Conscientiousness, Negative Emotionality, and Open-Mindedness; (2) Social Interaction Anxiety Scale (SIAS; Mattick and Clarke, 1998): The SIAS measures the degree of psychological distress when interacting with other people and provides a cutoff score for Social Anxiety (i.e., generalized irrational fears across numerous social situations with avoidance and impairments) (3) Differentiation of Self Inventory (DSI; Skowron and Friedlander, 1998): A measure of the ability to experience intimacy with and independence from others; the test includes the subscales Emotional Reactivity (i.e., the degree to which a person responds to environmental stimuli with emotional flooding, emotional lability, or hypersensitivity), Emotional Cutoff (i.e., feeling threatened by intimacy and feeling excessive vulnerability in relationships with others), I-Position (i.e., presence of a clearly defined sense of self and the ability to thoughtfully adhere to one’s convictions when pressured to do otherwise), and Fusion with Others (i.e., emotional overinvolvement with others, including triangulation and overidentification with parents); (4) Experience in Close Relationships (ECR-RD12; Wei et al., 2007): Assesses individual differences with respect to Attachment-related Anxiety (i.e., the extent to which people are insecure vs. secure regarding the availability of and responsiveness to the people they are romantically involved with) and Attachment-related Avoidance (i.e., the extent to which people feel uncomfortable being close to others vs. secure in depending on others); and (5) Vulnerable Attachment Style Questionnaire (VASQ; Bifulco et al., 2003): This instrument provides a total measure of the Vulnerability of Attachment including the subscales Insecurity of Attachment (i.e., feelings and attitudes relating to discomfort with, or barriers to, closeness with others, including inability to trust and hurt or anger at being let down) and Proximity Seeking (i.e., dependence on others and approach behavior).

All instruments are listed in Table 1.

### 2.4. Study design

The study was designed following a mixed-methods approach by which qualitative data as the primary source of data were expanded with quantitative data. This design allowed us to fully exploit the breadth of content and scope by using different methods for different parts of the study (Kuckartz, 2014). Therefore, we aimed at characterizing the phenomenon of interest based on the qualitative data and supplementing it with the results of psychometric testing to also situate it in existing psychological discourses.

The two parts of the study were conducted in the following order: (i) interview and (ii) psychometric testing. Interviews were conducted for each participant individually with two interviewers (T.D. and L.W.). Due to the security measures in place at the time of the data collection caused by the Covid-19 pandemic, the interviews were conducted online using the video meeting software Microsoft Teams (Microsoft 365, Version 1.5.00.22362). Video and audio channels were used for the entire meeting by all parties. Audios were recorded using an external digital voice recorder (Olympus WS-853, Tokyo) placed next to the interviewers’ computers. The video was not recorded. Participants were informed about the audio recordings prior to the start of the interviews. Participants were also informed about the general aim of the study and the interview procedure, stating that the interview will address how people with alexinomia feel and how it affects everyday life. Additionally, participants were informed about the GDPR-conform processing of personal data. Raw data audio files were kept on secure storage media at the university and stored locally and separately from any identifying personal data. Participants gave written consent to all described procedures.

The interviews lasted 32–61 min, with a mean duration of 42.2 min. For the second part of the study, the link to the questionnaire was provided by email. The online questionnaire was realized using SoSci Survey (Leiner, 2019) and was made available to participants on the website https://onlinebefragungen.sfu.ac.at/. It included five standardized psychological instruments (Table 1). There were 160 items in total; completing the questionnaire took about 60 min. All participants fully completed all parts of the study. All procedures were approved by the Sigmund Freud University (SFU) ethics committee.

### 2.5. Data analysis

#### 2.5.1. Analysis of qualitative data

The interviews were transcribed word for word. Content analysis focused on summarizing the main topics directly uttered, therefore, dialect and intonation were not transcribed. All interviews were conducted and analyzed in German. Participants’ quotes presented as text examples in this paper were translated from German to English by the authors for publishing purposes. Translations were made literally, meaning that the translation was kept as close as possible to the original wording but that the grammar rules of the target language (English) were applied. Personal data (i.e., names, places, etc.) were replaced with pseudonyms.

The interview data were coded according to Mayring’s procedure of an inductive, summarizing content analysis (Mayring, 2014, 2015). Mayring’s methodical concept is particularly useful at the interface with quantitative methods to enable the triangulation of qualitative and quantitative data. The procedure employs a hermeneutical logic in assigning categories to text passages, resulting in a hierarchical category scheme of main categories and subcategories of multiple order. This procedure aims at reducing a large body of data to its relevant thematic constituents, identifying a content structure, which can unravel and display the different layers of a phenomenon.

The inductive, summarizing coding process was structured into the following seven steps (Mayring, 2014): (1) Defining the category, (2) paraphrasing all utterances of all interviews that were relevant according to category definition, (3) generalizing the paraphrases to their main content, (4) first reduction, (5) second reduction, (6) listing all identified categories in the form of a category scheme, and (7) revising the category scheme including repetition of steps 2–5 in case the categories and codes did not work for additional data.

Further, the category scheme was summarized by revising the list of categories and generalized again by combining categories with similar meanings, thus identifying main- and subcategories. This step was repeated multiple times to carve out the main factors of the phenomenon.

The category definition focused on the identification of all relevant intra- and inter-personal factors characterizing the phenomenon of having difficulty with saying names in everyday practice including interactions where it occurs and their subjective experience, coping strategies when it happens, and self-theories on the development of the difficulty throughout the lifespan. To ensure reliability, two researchers coded the first interview. From this first interview, the initial main categories as well as the subcategories were created and on this practice the coding guidelines for the analysis of further interviews were developed. According to these guidelines, all other interviews were coded independently by two researchers.

#### 2.5.2. Analysis of quantitative data

To analyze the questionnaire data, participants’ test scores were subjected to a series of summary independent-samples *t*-tests, one for each subscale of each instrument for which norm data (i.e., sample sizes, means, and standard deviations) were available. These instruments were the BFI-2, DSI, and ECR-RD12. Questionnaires using cutoff-scores to indicate the presence or absence of a psychological trait (i.e., SIAS and VASQ) were analyzed separately for each subscale using one-sample *t*-tests tested against these respective cutoff values. The alpha-level was set to 0.05 for all analyses. All *p*-values were corrected for multiple comparisons (Holm-Bonferroni) across the entire data set. Hedges’ *g* is reported for all analyses as a measure of effect size.

## 3. Results

### 3.1. Qualitative data

We identified the following four main categories that, based on our data, constitute alexinomia: (1) Factors of subjective experience when trying to address others by their personal names such as emotional states and physical reactions, (2) general characteristics of alexinomia such as first occurrences, affected relationships, and affected forms of communication, (3) effects on relationships and on communication in relationships and strategies to cope, (4) vulnerability factors based on biographical information and current relationship patterns. Table 3 shows the complete category scheme in detail. In the following, the main categories are presented with significant quotes from the interviews to support our conclusions. In addition, the most important subcategories are elaborated to detail the phenomenon.

**TABLE 3:** Category scheme.

Main category	First level subcategory	Second level subcategory	Third level subcategory
A Subjective experience	A1 Negative emotions	A11 Anxiety/panic A12 Shame/embarrassment A13 Nervousness/restlessness A14 Discomfort A15 Regret/frustration A16 Perplexity/confusion/lack of understanding A17 Stress/restriction A18 Pressure/pressure to perform A19 Anger A110 Rumination A111 Failure/overload/inability A112 Effort	–
	A2 Negative self-concept	A21 Impolite A22 Inadequate/inappropriate A23 Strange/odd/funny/not normal A24 Crazy A25 Socially insecure A26 Not worth it A27 Quiet A28 The only one	–
	A3 Expectations and perceived functions of saying names	A31 Expected effect on others/what names stand for	A311 Seems forced/stupid/funny A312 Creates the feeling of being the center of attention A313 Triggers a negative reaction in the other person A314 Creates the feeling of being caught out A315 Feeling of being caught red-handed A316 Pronunciation could be wrong/one could make a mistake A317 Creates seriousness/strictness A318 Stands for respect A319 Stands for the whole person A3110 Has a magical quality A3111 Stands for identity/uniqueness A3112 Is invasive A3113 One is seen A3114 Could feel distant, formal, unemotional A3115 One shows oneself/makes oneself vulnerable A3116 Is personal/intimate A3117 Is beautiful A3118 It might disturb/distract the person A3119 It feels trivial/not special A3120 It could sound very emotional
		A32 Social functions	A321 Creates (too much) closeness/connection A322 Creates the feeling of being at the mercy of others A323 Dissolves closeness (when I say your name, I am someone other than you; dissolves symbiosis) A324 Establishes a boundary
	A4 Possible benefits of not (!) saying names	A41 Preserves something of one’s own A42 Protects from being hurt A43 Preserves a boundary/creates distance A44 Serves to express aggression A45 Serves to express repressed anger	–
	A5 How it feels to try to address someone by their name	A51 As if you were holding your breath A52 Blockage/inhibition/overcoming A53 Pausing A54 A little shock A55 Crossing a border A56 Feeling physically bad A57 Nausea A58 Chest area contracts A59 Feelings need to be turned off A510 Like looking someone in the eyes A511 Like physical contact A512 An inside-verbalization that cannot come out	–
B General characteristics	B1 Affected relationships	B11 Romantic relationships B12 Father/stepfather B13 People with funny names (e.g., names that are difficult to pronounce) B14 Men B15 In (almost) all relationships B16 Parents B17 Strangers B18 People with beautiful names B19 Colleagues in education B110 Adults/older people B111 Teachers/lecturers B112 Close/people known for quite some time/friends/important persons B113 Persons of authority B114 Very specific people	–
	B2 Affected forms of communication	B21 Personal contact B22 When saying first names B23 In serious situations B24 In conversation with third parties B25 In writing B26 In direct conversation	–
	B3 Using nicknames	B31 Nicknames are not used in affected relationships B32 Nicknames are used in non-affected relationships B33 Nicknames are used in affected relationships	–
	B4 Relation to one’s own name	B41 No identification with own name B42 Negative attitude toward own name B43 Pleasant when others say own name B44 Unpleasant when others say own name B45 One’s own name stands for punishment/annoyance B46 Being called by one’s own name creates distance B47 Being addressed by one’s own name creates an inferior position	—
	B5 Frequency (first occurrence, occurrences, duration, etc.)	B51 The difficulty always occurs in the affected relationships B52 The difficulty occurs over the entire duration of an affected relationship (e.g., throughout marriage) B53 The difficulty occurs consistently since the first romantic relationship B54 As early as kindergarten B55 In adolescence B56 Always B57 It is the normal state	–
	B6 Non-affected relationships/situations	B61 Interactions with women B62 With friends/colleagues/acquaintances (male and female) B63 Animals B64 Siblings B65 Formal relationships B66 Parents B67 With new acquaintances B68 At sports B69 With particularly good friends B70 With children	–
	B7 Non-affected forms of communication	B71 In Conversation with Third Parties B72 In writing B73 In playful situations	–
C Effects and coping strategies	C1 Effects on making contact	C11 Is difficult C12 Long waiting times until contact is made/conversation begins C13 None (because coping strategy works so well) C14 No contact C15 Attempt to attract attention “telepathically”	–
	C2 Effects on affected relationships	C21 Noticed by others C22 Unnoticed by others C23 Perceived as impersonal/cold/distant C24 Offends/hurts/makes people sad C25 Not understood C26 Creates a barrier/distance C27 Some things remain unsaid C28 Mistrust arises C29 No significant influences	-
	C3 Coping strategies	C31 Starting a conversation without address C32 Establishing contact through eye contact C33 Use of impersonal forms of address (hey, etc.) C34 Making contact by touching (e.g. tapping on the shoulder) C35 Masking/avoidance C36 Attempt to say names (unsuccessful) C37 Use of nicknames C38 Use of surnames C39 Use of text messages instead of face-to-face conversation C310 Saying names in a funny way/with dialect/as a joke C311 Joking about it	–
	C4 Intervention/therapy	C41 Talking about it (with affected people) C42 Psychotherapy C43 Trying to break through the problem C44 Exercise/dry training C45 Fear of therapy C46 Researching the topic	–
D Vulnerability factors	D1 Childhood and family	D11 Early signs of social anxiety (e.g., shyness, frequent blushing, etc.) D12 Trauma/neglect/parental abuse/violence D13 Mental disorders in the family (e.g., depression, addiction, narcissism, etc.) D14 Absent parent (e.g., early death of a parent, absent father, etc.) D15 Unstable family relationships (e.g., separation, divorce, strongly changing caregivers) D16 Hardly any or no contact with family members D17 Conflict avoidance in childhood/birth family D18 Little communication/openness in the family D19 Distant relationship with family D110 Psychological problems in childhood D111 Good family relationships in childhood D112 Few friendships/bullying D113 Dispute in the family D114 Pressure to perform	–
	D2 Current relationship patterns	D21 Conflict avoidance D22 Difficulty perceiving and communicating one’s own boundaries D23 Dependent relationships D24 Symbiotic relationships, desire to merge D25 Difficulty trusting/relating/jealousy D26 Few/no positive relationships D27 Few male friends D28 Good/happy/trusting current relationship D29 Low self-worth	–
	D3 Difficulty in the expression of emotions	D31 Difficulty expressing feelings verbally (general) D32 Difficulty expressing affection verbally D33 Difficulty expressing gratitude verbally D34 Difficulty expressing aggression D35 Difficulty expressing needs	–

#### 3.1.1. Factors of subjective experience

All participants in the study reported having problems with addressing persons by their names. When, for instance, asked to describe the problem in their own words, they said:

It has always been like that, as long as I can remember, in kindergarten, in school. I couldn’t address classmates with their names, and it took extreme efforts to try. I used to think twice about whether I really needed to say the name, whether my question was important enough or not. (…) I then became really conscious of it, when I met my husband about one and a half years ago. I wanted to address him by name, but I could not do it. I wanted to, but I couldn’t. That’s when I realized that it’s a problem, that I can’t say other people’s names. (…) I tried to practice his name alone, because I thought maybe it’s because I’m afraid that I pronounce it wrong or something like that, and I still couldn’t do it. And yes, even to this day it’s still difficult for me to address him by name, I always say “you” or “hey,” things like that (Participant 3).
It’s very difficult for me to call someone by name, but also when I talk about someone, to say the name. (…) I always say “I was walking with my neighbor” or “I meet with my fellow student.” I wouldn’t say their first names. That’s when I really notice how difficult it is for me, no matter whether it’s a good friend or not such a good friend, to say the name. (…) To give an example, I was helping in the field to dig up potatoes. We were up at the back of the harvester and my ex-boyfriend was driving the tractor. And then the ladies who were also helping asked me to please tell him to slow down, otherwise it’s going too fast. I was completely overwhelmed, and I shouted “Hey, hey, hey!”, because I just could not call the first name. He didn’t hear me and finally one of the other ladies then said his first name and he then heard that and stopped. So that was a situation where they actually hoped for me to tell him to please slow down. But I just couldn’t do it, I just didn’t call his name, I said “Hey, hey!”, I was like, I failed (Participant 8).

in situations in which saying a name is intended, participants frequently reported experiencing negative emotions such as anxiety/panic, shame/embarrassment, regret/frustration, and feelings of inadequacy/failure. These feelings are often accompanied by unpleasant physical reactions that, for example, were described as “a tight feeling in the chest” (Participant 10) and were compared to how it feels to “touch someone” (Participant 11) and “to look someone into the eyes” (participant 11). In the words of participant 2:

It feels almost a bit like the beginning of a panic attack. Like loss of control and nervousness, agitation (…) a feeling of discomfort, an “I’m about to be the center of attention” feeling or like when you have to give a lecture, like stage fright, really extremely uncomfortable and scary. It’s insecurity. Insecurity (Participant 2).

Participants further highlighted what seems to be a complex relationship of the inability to say a name and the control of closeness and distance in a (often romantic) relationship. While about half of our participants reported experiencing forms of alexinomia in all their social relationships (7 participants), several participants reported that the problem became more severe the closer the relationship (4 participants) and many claimed that it is strongest in romantic relationships (10 participants). Saying the name of a loved one was frequently described as too close, too personal, too intimate, and thus overwhelming and emotionally exposing. Therefore, some affected individuals speculated that not saying a name could have the function of keeping the other person at a certain distance. This distance, however, puts strain on the relationship and affected individuals expressed a strong desire to be able to overcome it. Almost contrary to the above situations, for some participants using names was also described as distancing and impersonal, as if using a partner’s personal name would take away “the magic” (participant 4) between them and thus create an unwanted distance. Along these lines, some participants indicated the use of names deliberately to control the distance in a relationship. Participant 10 described the dilemma of a deep longing for closeness and worries about the vulnerability that would come with it as follows: “I’m not sure if I would be able to cope with this, this closeness that would then be there. The vulnerability that would then be there, although this is what I want so badly.” (Participant 10).

Participant 12 addressed the complexity of the impact of naming on the experienced closeness or distance in social relationships:

It’s strange, at first I thought that somehow it had something to do with closeness, but then I realized that on the other hand you can use it very well to maintain distance. It’s somehow this autonomy, closeness, distance, where that plays a big role, especially with hierarchies, like bosses or superiors, or in love relationships, where you’re kind of afraid of showing yourself vulnerable, where you think “oh, there is suddenly someone above yourself.” (…) There is also a bit of fear that it sounds funny for him because I might pronounce it somehow special or that he could hear my emotions because of me pronouncing it somehow differently. (…) To me, saying his name would feel closer, but when he says my name, I feel more of a distance. Because everybody calls me that. It’s nothing special (Participant 12).

#### 3.1.2. General characteristics

Affected relationships included mainly romantic relationships, close friends, and family. However, several participants reported experiencing alexinomia in all relationships, regardless of the level of closeness. Hierarchy seems to play a role in a way that the symptoms are more severe in interactions with persons of authority such as bosses, superiors, and teachers. For example:

At school it was sometimes unpleasant to address teachers by name, especially those who were very authoritarian. (…) I couldn’t say the names of people who were hierarchically superior. (…) In relationships with a power dynamic it is more pronounced (Participant 7).

Some participants indicated that the severity of the problem also depends on the name in question. Unusual names, names that are difficult to pronounce, and names that are considered particularly beautiful or unattractive can make the symptoms more severe.

Other factors that contribute to the name saying difficulties of our participants included the relationship to one’s own name, whether one likes to be addressed by name or not, and the general attitude toward naming others and being named by others. Most participants said that they liked to be addressed by name and that they considered it a sign of respect. Some participants, however, associated being called by the name with a certain seriousness that feels strict, cold, and generally unpleasant. Interestingly, even in very severe cases of alexinomia, there were usually certain individuals whose names could be said (e.g., the name of a pet animal or of a sibling).

Frequently, the symptoms of alexinomia extend to conversations with others about the person with the problematic name. For some participants however, the symptoms were limited to only direct personal conversations. Participant 1 described the situation of saying her partner’s name when talking to someone else about her partner while him being present: “With very great restraint and with a low tone, it works. But so that he almost doesn’t hear it. It’s unpleasant, but I somehow manage.” (Participant 1) In conversations with others, names are often avoided even if the respective person is absent by refering to their social role such as “my friend,” “my cousin,” etc. Symptoms can be less severe in written compared to verbal direct communication. About half of our particpants have no difficulty writing someone’s name in Emails and texts. For participant 1 it “just feels safer in writing.” Other participants on the other hand indicated avoiding names also in writing, for example participant 13 who prefers to use nick names, such as “sweetheart” to greet a female friend in a written conversation.

Characteristically the signs of alexinomia were there from the very beginning of the affected relationships and, if untreated, were present throughout the entire duration of the relationship. Most of our participants reported that they recall having the problem since they were children or teenagers or that it “has always been there” (participant 11). In several cases, participants first noticed having the problem in their first romantic relationship.

#### 3.1.3. Effects and coping mechanisms

Alexinomia affects communication in relationships in a multitude of ways. Instead of using names, most affected individuals reported starting a conversation without using a personal address or by using an impersonal address such as “Hey” or “Hi.” Often, they would wait for some time before saying something until they got the addressees’ attention *via* eye contact, physical touch (e.g., tapping on the shoulder) or using “telepathy” (Participant 10). If none of these worked, several participants reported that they would rather stay silent and forget about the intended conversation than using the name. To give an example:

I wait for eye contact. When sitting at a large table with many people talking, it’s sometimes a bit difficult. I really have to wait until he looks directly at me and then say, “Can you please pass me something?”. So stupid. Or just “You” or “Hey,” so rude actually. Or I touch him, tap him and just start talking. (…) Anyway, apparently I have done really well, no one has noticed until now (Participant 2).

According to some participants, it sometimes helps to use nicknames or to say the name in a playful way (4 participants). Most participants, however, said that using nicknames was also not an option for them (9 participants).

While these compensatory strategies can help avoiding names and hiding the problem successfully, alexinomia can put a serious strain on a relationship due to the distance and insecurities that it facilitates in both partners:

With my husband, I notice that it burdens him. (…) He spoke to me directly about it and said that he thinks it’s a shame that I never address him by his name. He said that it annoys him and that he finds it impersonal and kind of disrespectful, like when you’re not looking at each other in a conversation. That’s how it feels, like I’m looking away. I then tried to explain to him, it’s just the opposite. (…) It is very hard for me to realize that he feels offended and put down. We have very, very different views on what it means that I am not saying his name (Participant 1).
When my partner confronted me, that was actually the most unpleasant thing, it was like getting caught, “Oh my God, now someone has caught me, after all these years someone has now really caught me.” Yes, that was the most unpleasant thing. (…) It was very, very, very embarrassing, I felt ashamed that I had to admit having a problem with this. It was a feeling of extreme shame (Participant 2).
It burdens me, because I see that it burdens him. Because he says that he is also a normal person, like me, and that he also needs love and to hear his name from my mouth and that he never gets that from me. I get it from him, but he does not get it (Participant 6).

#### 3.1.4. Vulnerability factors

The main purpose of this study was to describe alexinomia with regards to its main characteristics and attributes, thus the underlying causes of the problem will be the subject of future research. However, there were details that were mentioned multiple times in the interviews that could constitute factors of vulnerability which might contribute to the development and maintenance of alexinomia. From the category of biographical details participants mentioned early signs of social anxieties as a child (e.g., shyness; blushing; etc.), childhood trauma, violence, and neglect, and the presence of psychological disorders such as depression, addiction, and personality disorders in the family. From the category of current relationship patterns participants displayed a tendency to avoid conflicts and to enter symbiotic relationships and to have low levels of self-esteem and self-confidence in relationships and social interactions. Another category was formed from reports of having difficulty in expressing emotions. Participants reported having problems with expressing emotions in general and especially with verbally expressing love, affection, and gratefulness (3 participants).

### 3.2. Quantitative data

All participants completed all questionnaires. There were no missing data and no outliers. Therefore, all available data was included in the analysis.

#### 3.2.1. Big Five personality traits (BFI-2)

**FIGURE 1:**
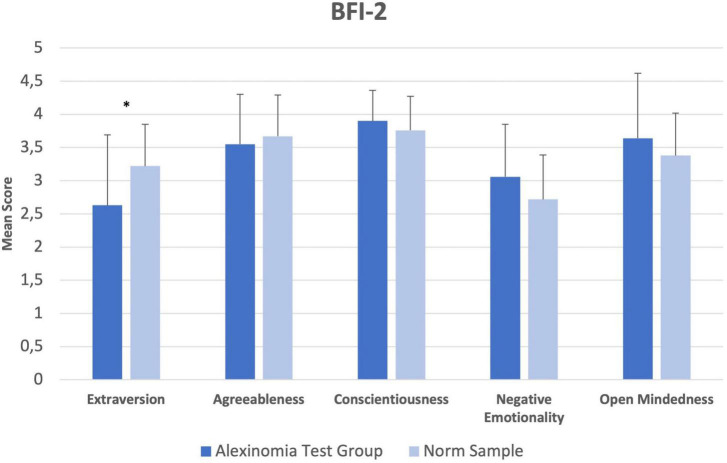
BFI-2. Comparisons of group means in the BFI-2. Error bars indicate 1 SD. *p < 0.05.

Summary independent-samples *t*-tests were calculated for each of the five subscales of the BFI-2. The mean test score of the test group was significantly lower in the scale *Extraversion* (*M* = 2.63; *SD* = 1.06) compared with norms (*M* = 3.22; *SD* = 0.63), *t*(781) = -3.302, *p* = 0.014, *g* = 0.92. Comparisons of the other subscales were not significant (Figure 1).

#### 3.2.2. Social anxiety (SIAS)

**FIGURE 2:**
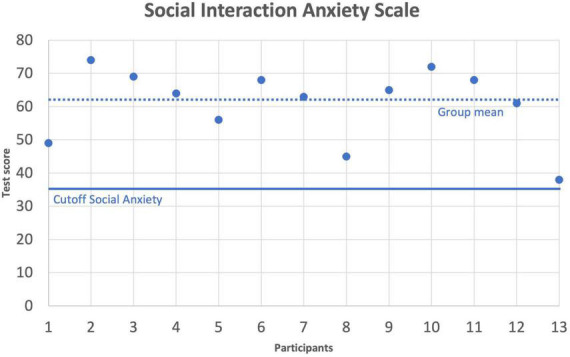
Social Interaction Anxiety Scale (SIAS). Individual scores and group mean (dotted line) in the SIAS. The solid line indicate the SIAS cutoff value for social anxiety.

To analyze data related to social anxiety as measured by the SIAS, participants’ test scores were compared with the cutoff value for social anxiety. All participants scored above the cutoff of 36 which indicates the presence of social anxiety (Mattick and Clarke, 1998). The group mean in the SIAS was *M* = 60.92 (*SD* = 10.94). A one-sample *t*-test against the test value of 35 confirmed a significant group effect for social anxiety, *t*(12) = 8.547, *p* < 0.001, *g* = 2.22 (Figure 2).

#### 3.2.3. Differentiation of self (DSI)

**FIGURE 3:**
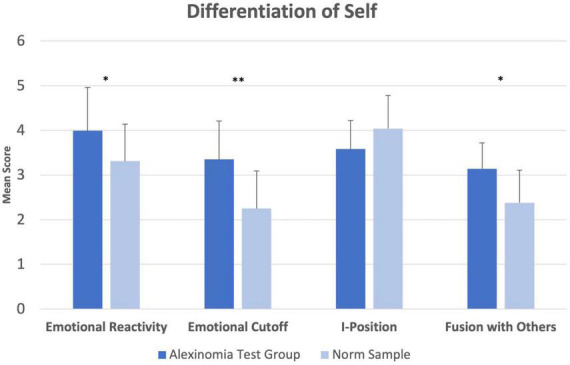
Differentiation of Self Inventory (DSI). Comparisons of group means in the DSI. Error bars indicate 1 SD. *p < 0.05, **p < 0.1.

The four scales of the DSI were analyzed using summary independent-samples *t*-tests. Group means were compared to norm data. This comparison revealed significantly higher scores of the test group compared with norms in the following scales: *Emotional Reactivity* (*M* = 3.99; *SD* = 0.97 vs. *M* = 3.31; *SD* = 0.83; *t*(270) = 2.859, *p* = 0.035, *g* = 0.82), *Emotional Cutoff* (*M* = 3.35; *SD* = 0.87 vs. *M* = 2.25; *SD* = 0.84; *t*(270) = 4.600, *p* = 0.010, *g* = 1.31), and *Fusion with Others* (*M* = 3.14; *SD* = 0.58 vs. *M* = 2.38; *SD* = 0.73; *t*(74) = 3.525, *p* = 0.011, *g* = 1.07). Comparisons of the subscale *I-Position* were not significant (Figure 3).

#### 3.2.4. Experience in close relationships (ECR-RD12)

**FIGURE 4:**
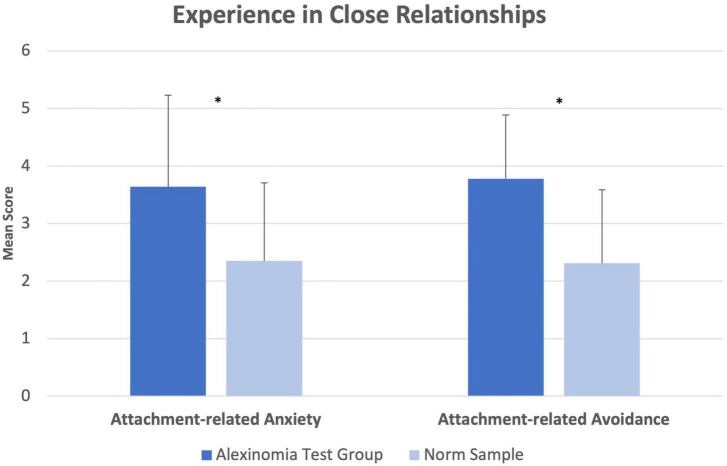
Experience in Close Relationships (ECR-RD12). Comparisons of group means in the ECR-RD12. Error bars indicate 1 SD. *p < 0.05.

Our sample differed significantly from norms in both scales of the ECR-RD12, *Attachment-related Anxiety* (*M* = 3.64; *SD* = 1.59 vs. *M* = 2.35; *SD* = 1.36; *t*(260) = 3.306, *p* = 0.013, *g* = 0.94) and *Attachment-related Avoidance* (*M* = 3.78; *SD* = 1.11 vs. *M* = 2.31; *SD* = 1.28; *t*(260) = 4.060, *p* = 0.012, *g* = 1.16) as revealed by summary independent-samples *t*-tests (Figure 4).

#### 3.2.5. Vulnerability of attachment (VASQ)

**FIGURE 5:**
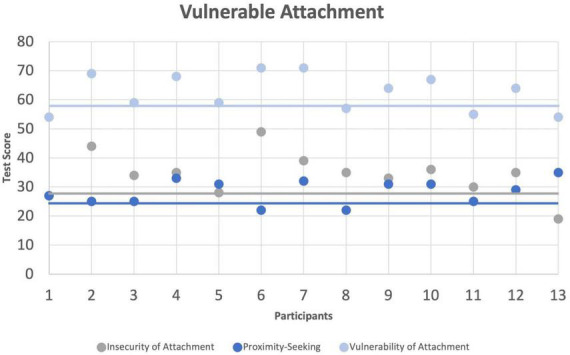
Vulnerable Attachment Style Questionnaire (VASQ). Individual scores in the VASQ. Lines indicate VASQ cutoff values for the respective scales.

The VASQ includes three scales, and it offers cutoff values for each of these. In our sample, 10 out of 13 participants scored above the cutoff of 57 which indicates the presence of general *Vulnerability of Attachment*. The cutoff for the scale *Insecurity of Attachment* of 30 was met or exceeded by 10 participants and eights participants scored 27 or higher in the scale *Proximity Seeking* (Figure 5). One-sample *t*-tests against the respective test values of each scale showed a significant group effect for *Vulnerability of Attachment*, *t*(12) = 3.619, *p* = 0.032, *g* = 0.94. Differences in the other scales were not significant (*p*s > 0.05).

Table 4 shows a summary of the results of the quantitative analysis of questionnaire data.

**TABLE 4:** Quantitative analysis of questionnaire data.

Scale	Test sample		Norm sample		*t*	df	*P* (corr.)	Effect size (Hedges’ *g*)
	***N***	***M* ± *SD***	***N***	***M* ± *SD*/Cutoff**				
**BFI-2**								
Extraversion	13	2.63 ± 1.06	770	3.22 ± .63	-3.302	781	0.014*	0.92
Agreeableness	13	3.90 ± 0.46	770	3.76 ± .51	0.983	781	>0.05	–
Conscientiousness	13	3.55 ± 0.75	770	3.67 ± .62	-0.690	781	>0.05	–
Negative emotionality	13	3.06 ± 0.79	770	2.72 ± .67	-1.781	781	>0.05	–
Open mindedness	13	3.64 ± 0.98	770	3.38 ± .64	1.438	781	>0.05	–
**SIAS**								
Social anxiety	13	60.92 ± 10.94	–	36	8.547	12	>0.001***	2.22
**DSI**								
Emotional reactivity	13	3.99 ± 0.97	259	3.31 ± .83	2.859	270	0.035*	0.82
Emotional cutoff	13	3.35 ± 0.87	259	2.25 ± .84	4.600	270	0.010**	1.31
I-Position	13	3.58 ± 0.64	259	4.04 ± .74	-2.199	270	>0.05	-
Fusion with others	13	3.14 ± 0.58	63	2.38 ± .73	3.525	74	0.011*	1.07
**ECR-RD12**								
Attachment-related anxiety	13	3.64 ± 1.59	249	2.35 ± 1.36	3.306	260	0.013*	0.94
Attachment-related avoidance	13	3.78 ± 1.11	249	2.31 ± 1.28	4.060	260	0.012*	1.16
**VASQ**								
Vulnerability of attachment	13	62.46 ± 6.44	–	56	3.619	12	0.032*	0.94
Insecurity of attachment	13	34.15 ± 7.55	–	29	2.462	12	>0.05	-
Proximity seeking	13	28.31 ± 4.25	–	26	1.958	12	>0.05	-

All *p*-values were corrected for multiple comparisons (Holm-Bonferroni).

*p < 0.05, **p < 0.01, ***p < 0.001.

To summarize, results from psychometric testing show that our sample of individuals affected by alexinomia fulfilled the criteria for the presence of social anxiety both on individual and group levels. In addition, participants scored significantly higher on several attachment- and relationship-related anxiety and vulnerability scales compared with the respective norms. The ability to regulate emotions was reduced and participants showed decreased levels of extraversion. This pattern of results is well in line with previous research showing that social anxiety is related to insecure attachment (Manning et al., 2017), impaired regulation of emotions (Jazaieri et al., 2015), and negatively correlated with extraversion (Kaplan et al., 2015).

Taken together, and as a very first attempt to conceptualize alexinomia from a clinical diagnostics point of view, data from both qualitative and quantitative analyses suggest the following key symptoms of alexinomia: (i) The individual experiences fear or anxiety in situations, in which using personal names is intended; (ii) Intending to use a name almost always provokes fear or anxiety in the individual in at least one relationship; (iii) the feared situations are avoided or dealt with using compensatory strategies, (iv) the fear, anxiety, or avoidance is persistant; (v) the fear, anxiety, or avoidance causes significant distress or impairment in social, occupational, or other important areas of functioning.

This preliminary symptom list is based on the diagnosis of Social Anxiety Disorder according to the *Diagnostic and Statistical Manual of Mental Disorders* (DSM-5), is not exhaustive and should be seen as a first proposal to the scientific community to inspire the generation of new hypotheses to testify the construct in a more rigorous statistical fashion.

## 4. Discussion

The aim of the study was to explore and describe the distinctive features of alexinomia—a previously unknown and scientifically undocumented psychological phenomenon characterized by an inability to use personal names in communication. Using a mixed-methods approach combining qualitative data from personal interviews with participants who are personally affected by alexinomia, and quantitative data gained from psychometric testing of the same sample, our findings allowed us to describe alexinomia on a phenomenological level and to get an understanding of its key attributes and potential links to other psychological constructs including social anxiety and attachment.

Results indicated that all participants had the desire to use personal names in everyday social interactions, especially in their closest relationships. The phenomenological experience of alexinomia, however, is that of an inability to do so. This inability is accompanied by feelings of anxiety, panic and physical as well as psychological discomfort. The affected individuals’ experience is further characterized by feelings of regret, embarrassment, and shame, especially when confronted with their behavior by others.

Our data suggest that alexinomia usually occurs for the first time in childhood or adolescence and is then ongoing. It affects all forms of relationships and communication and is strongest in romantic relationships and in direct verbal communication. Interestingly, the problem becomes increasingly severe the closer the relationship, which often puts a heavy strain on also the romantic relationships of the people affected. There operates a painful ambiguity in individuals affected by alexinomia in the form of a longing for closeness and intimacy in a relationship on the one hand and feelings of vulnerability and being emotionally overwhelmed, that come with saying a loved ones’ name on the other hand. Such feelings of emotional exposure and fear of unbearable closeness seem to constitute the core features of the name saying avoidance behaviors described here.

Results based on the analysis of psychometric tests suggest a strong link between alexinomia-related behaviors and social anxiety (Stein and Stein, 2008). All participants of our sample fulfilled the criterion for the presence of social phobia according to the Social Interaction Anxiety Scale (Mattick and Clarke, 1998). This finding is well in line with the main findings from the interviews showing that feelings of anxiety and embarrassment are among the main symptoms of alexinomia. Social anxiety has been shown to be linked to low intimacy and decreased satisfaction in romantic relationships (Davila and Beck, 2002; Rodebaugh, 2009) and, thus, can also be conceptualized from an attachment viewpoint. Participants in our study displayed increased levels of attachment-related anxiety and avoidance as measured by the ECR-RD12 and vulnerability of attachment as measured by VASQ. These findings indicate that those affected by alexinomia are insecure with regards to the availability of and responsiveness to the people they are romantically involved with and tend to feel uncomfortable being close to others. Moreover, measures of the ability to experience intimacy with and independence from others, indicated increased levels of emotional reactivity (i.e., emotional flooding, emotional lability, or hypersensitivity), increased levels of feeling threatened by intimacy, and an emotional overinvolvement with others, including triangulation and overidentification with parents. Additionally, our sample showed significantly lower levels of extraversion compared with norms as measured by the BFI-2. These results from standardized psychological instruments are well in line with the qualitative data gained from the interviews.

Taken together our findings based on both qualitative and quantitative data suggest alexinomia lies at the center of where social anxiety, fear of attachment, and impaired emotional processing with regards to social interactions meet.

Several additional factors that relate to and/or potentially interact with alexinomia were observed but not elaborated in depth in the present study. These included participants’ attitudes toward their own names, whether they liked their name and being called by it or not and its co-existence with related phenomena of impaired communication, including an inability to call one’s parents “Mom” and “Dad” and to say “I love you.” So far, we do not have any data on whether or not affected individuals are able to say and write their own names. Another potentially directly related concept is name idealization in in-love couples, which, together with name avoidance, has been suggested to be a sign of the partners’ name being a taboo (Leisi, 1980).

Alexinomia should be differentiated from physical and cognitive impairments that affect the ability to produce language, known as aphasia. Also, alexinomia is not caused by impaired memory for names. Further, deliberately refusing to say someone’s name to humiliate a person or to show dominance or disrespect, in our opinion, is also fundamentally different from the type of name avoidance described here.

Our sample consisted of German-speaking women aged between 18 and 40 years, therefore all conclusions drawn from this study are limited to this sociocultural group, which was selected based on the availability of contacts of affected individuals. With these limitations in generalizability of our findings in mind, findings from ongoing online research suggest alexinomia to be prevalent also in other sociocultural groups (unpublished data). Hence, future research will explore whether alexinomia also occurs in other genders, age groups, cultural regions and how it is affected by language and cultural aspects of naming and name usage in everyday language. A scale to measure the precence and severity of alexinomia-related symptoms is warranted for statistically validating the construct further and for the development of a clinical diagnostic. Future quantitative research on larger samples might further address the prevalence of alexinomia in the general population, its neurobiological foundations, and psychosocial origins and causes. Not at least, the research presented here was mainly focused on the impact of alexinomia on primarily affected individuals. However, our findings also suggest a significant impact on family and other affiliates; therefore, looking at these secondarily affected individuals will be another important next step in this line of research to further understand alexinomia and to work toward a potential treatment of it.

## Data availability statement

The raw data supporting the conclusions of this article will be made available by the authors, without undue reservation.

## Ethics statement

The studies involving human participants were reviewed and approved by Sigmund Freud University (SFU) Ethics Committee. The patients/participants provided their written informed consent to participate in this study. Written informed consent was obtained from the individual(s) for the publication of any potentially identifiable images or data included in this article.

## Author contributions

TD and LW collected the data. TD and NR analyzed the data. TD wrote the first draft of the manuscript. NR wrote sections of the manuscript. All authors contributed to the conception and design of the study, manuscript revision, read, and approved the submitted version.

## Footnotes

1
www.lisawelleschik.at
